# Predictive Value of Baseline Left Ventricular Global Longitudinal Strain for Cardiac Dysfunction in Patients with Moderate to High Risk of Cancer Therapy-Related Cardiovascular Toxicity

**DOI:** 10.3390/ph18101530

**Published:** 2025-10-11

**Authors:** Anna Borowiec, Patrycja Ozdowska, Magdalena Rosinska, Agnieszka Maria Zebrowska, Slawomir Jasek, Beata Kotowicz, Hanna Kosela-Paterczyk, Elzbieta Lampka, Zbigniew Nowecki, Jan Walewski

**Affiliations:** 1 Non-Commercial Clinical Trials Outpatient Clinic, The Maria Sklodowska-Curie National Research Institute of Oncology, 02-781 Warsaw, Poland; 2 Department of Computational Oncology, The Maria Sklodowska-Curie National Research Institute of Oncology, 02-781 Warsaw, Poland; 3 Unit for Screening Studies in Inherited Cardiovascular Diseases, The Cardinal Stefan Wyszynski National Institute of Cardiology, 04-628 Warsaw, Poland; 4 Cancer Biomarker and Cytokines Laboratory Unit, The Maria Sklodowska-Curie National Research Institute of Oncology, 02-781 Warsaw, Poland; 5 Department of Soft Tissue/Bone Sarcoma and Melanoma, The Maria Sklodowska-Curie National Research Institute of Oncology, 02-781 Warsaw, Poland; 6 The Gynecologic Oncology Clinic, The Maria Sklodowska-Curie National Research Institute of Oncology, 02-781 Warsaw, Poland; 7 Department of Brest Cancer and Reconstructive Surgery, The Maria Sklodowska-Curie National Research Institute of Oncology, 02-781 Warsaw, Poland; 8 Department of Lymphoid Malignancies, The Maria Sklodowska-Curie National Research Institute of Oncology, 02-781 Warsaw, Poland

**Keywords:** cardiotoxicity, anthracycline, cancer therapy-related cardiovascular toxicity, left ventricular peak systolic global longitudinal strain

## Abstract

**Background:** Anthracycline-based chemotherapy is associated with a risk of cancer therapy-related cardiac dysfunction (CTRCD), particularly in patients with moderate to high cardiovascular risk. Left ventricular global longitudinal strain (GLS) is a sensitive marker for early myocardial dysfunction, but the prognostic value of baseline GLS in this population remains unclear. **Objective:** We aimed to evaluate whether baseline GLS can predict CTRCD in moderate- to high-risk cancer patients undergoing anthracycline chemotherapy. **Methods:** In this prospective, single-center observational study, 80 anthracycline-naive cancer patients classified as moderate or high risk were enrolled. Baseline GLS was assessed via speckle-tracking echocardiography, with a threshold of ≥−18% considered decreased. Patients were followed for 12 months, and the primary endpoint was the development of CTRCD per ESC 2022 Cardio-oncology guidelines. **Results**: Of the 77 patients completing follow-up, 27.3% had decreased baseline GLS. CTRCD occurred in 62.4% of patients, with higher incidence among those with decreased GLS (76.7%) compared to those with normal GLS. In multivariable analysis, GLS ≥−18% was the only significant independent predictor of CTRCD (RR 12.0, 95% CI 2.0–71.9; *p* = 0.0065). All-cause mortality was also significantly higher in patients with decreased baseline GLS (19.1% vs. 1.8%, *p* = 0.018). **Conclusions:** Decreased baseline global longitudinal strain is an independent predictor of cancer therapy-related cardiac dysfunction and all-cause mortality in moderate- to high-risk patients receiving anthracycline therapy. These findings support the inclusion of baseline GLS in pre-treatment cardiovascular risk assessment, particularly in patients with an LVEF above 54%, to more effectively identify those who may benefit from early cardioprotective interventions.

## 1. Introduction

Cancer-related mortality has demonstrated a substantial decline in recent decades, largely attributable to improvements in early detection and the increased efficacy of contemporary chemotherapeutic regimens. Nonetheless, the cardiotoxic effects of these agents attenuate the overall therapeutic benefit [1]. One of the most significant cardiotoxic effects of chemotherapy is myocardial damage, which can result in heart failure and a reduction in left ventricular ejection fraction (LVEF), typically assessed using echocardiography or cardiac magnetic resonance imaging. Left ventricular global longitudinal strain (GLS), assessed through speckle tracking echocardiography, is a widely accepted method for detecting early changes in cardiac function, often identifying alterations before a decrease in LVEF. Multiple investigations in patients receiving chemotherapy have shown that a decline in global GLS generally precedes the clinical manifestation of heart failure [2,3,4,5].

In view of its established diagnostic and prognostic value, the measurement of GLS is now recommended as part of the routine baseline assessment and monitoring of patients receiving potentially cardiotoxic therapies. Current guidelines define a relative reduction of 15% or more in GLS as clinically significant [1]. In a retrospective study, baseline global longitudinal strain has been demonstrated to predict cancer therapy-related cardiovascular toxicity (CTRCD) in low-risk patients undergoing anthracycline therapy [6]. Prospective studies investigating the role of baseline GLS in predicting cardiotoxicity during anthracycline treatment in moderate- and high-risk patients remain limited.

### The Research Question

In oncological patients with moderate or high risk of cancer therapy-related cardiovascular toxicity who receive anthracyclines, does baseline left ventricular global longitudinal strain predict the development of cancer therapy-related cardiac dysfunction?

## 2. Results

### 2.1. Baseline Characteristics of the Patients

According to the study protocol, 80 patients were enrolled, of whom 77 (96%) completed follow-up with a mean observation period of 11.5 months. The mean age at baseline was 60.5 years, and 72 patients (93.5%) were female. During follow-up, five patients (6.5%) died: two from heart failure and three from cancer progression.

Most patients (*n* = 59, 76.6%) had breast cancer, whereas 11 (14.3%) had sarcoma and 7 (9.1%) had lymphoma. Based on current ESC cardio-oncology guidelines, 40 patients (51.9%) were classified as moderate risk and 37 (48.1%) as high risk for cancer therapy-related cardiovascular toxicity.

At baseline, the mean global longitudinal strain of the left ventricle was 19.1% (SD 2.0%), with decreased GLS (≥−18%) observed in 21 (27.3%) patients and ≥−16% in 10 (12.9%) patients. Additionally, the mean left ventricular ejection fraction (LVEF) for the entire group was 61.6%. The baseline characteristics of the patients are presented in Table 1.

### 2.2. Associations Between Global Longitudinal Strain of the Left Ventricle and Clinical Parameters

There were no significant differences in clinical characteristics between patients with normal and decreased GLS, except that those with decreased baseline GLS more frequently had NT-proBNP levels exceeding the upper limit of normal (ULN) as defined by the local laboratory reference range (*p* = 0.031), Table 1.

### 2.3. Incidence of CTRCD

**Figure 1:**
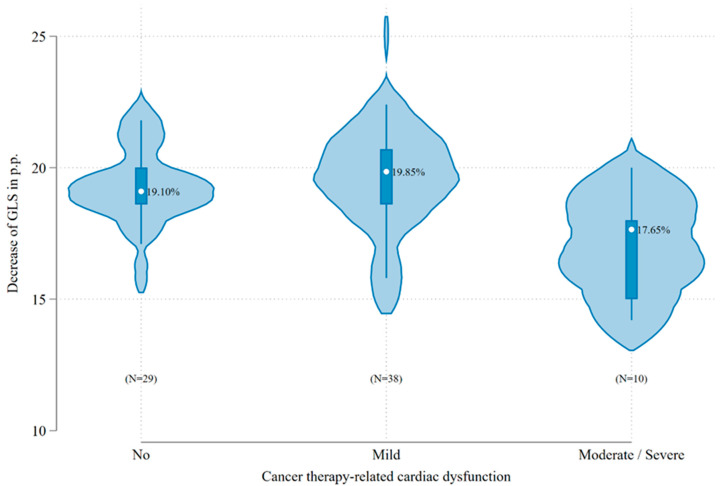
Distribution of baseline GLS (%) by severity of CTRCD. Dot: median value, box: interquartile interval, spikes: upper- and lower-adjacent values.

Overall, cancer therapy-related cardiac dysfunction was observed in 48 patients (62.4%). Mild CTRCD was the most frequent presentation, occurring in 38 patients (49.4%; 95% CI, 38.2–60.5%), followed by moderate CTRCD in 7 patients (9.1%; 95% CI, 4.3–18.0%) and severe CTRCD in 3 patients (3.9%; 95% CI, 1.2–11.6%). CTRCD was diagnosed in 14 (76.7%) of 21 patients with decreased baseline GLS. Furthermore, CTRCD occurred in 8 (80%) of 10 patients with baseline GLS ≥ −16%. The mean baseline GLS of patients who developed CTRCD during treatment with anthracyclines significantly differed from the mean baseline GLS of patients who did not have moderate/severe CTRCD (−17.1% vs. −19.4%; *p* = 0.002), Table 2. Figure 1 shows the distribution of baseline GLS values stratified by CTRCD severity.

### 2.4. Univariate and Multivariate Analysis

In univariable analysis we confirmed significant risk ratios of severe CTRCD for high-risk group vs. moderate-risk group (RR 4.3, 95% CI 1.0–19.1), LVEF <55% vs. ≥55% (RR 7.8, 95% CI 3.2–19.3), as well as increasing NT-proBNP (RR 1.8, 95% CI 1.0–3.3 per unit increase on log scale) and GLS reduction (RR 0.58, 95% CI 0.49–0.68 per unit increase).

Decreased baseline GLS was the strongest and at the same time the only significant predictor in the multivariable model (RR for ≥−18% vs. <−18%: 12.0, 95% CI 2.0–71.9, *p*-value 0.0065), Table 3. For the other covariates, effect estimates were directionally consistent with the univariable analyses but were imprecise, with confidence intervals overlapping the null.

### 2.5. Secondary End Point of All-Cause Mortality

The all-cause mortality rate was significantly higher in patients with decreased baseline GLS.

**Figure 2:**
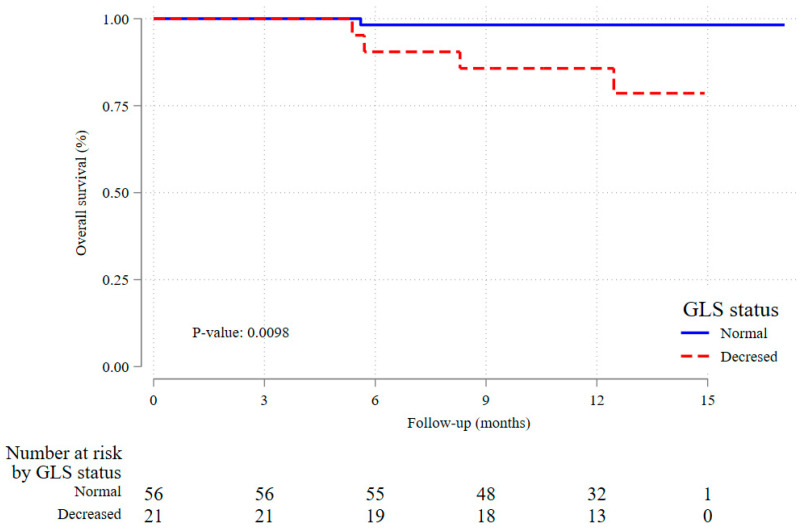
Overall survival by initial GLS status.

Survival in the 12-month observation period was 98.2% (95% CI 88.0–99.7%) among those with normal GLS as compared to 85.7% (95% CI 62.0–95.2%) in patients with decreased baseline GLS, Figure 2. These results suggest that decreased baseline GLS is strongly associated with increased all-cause mortality, highlighting its potential role as a prognostic marker.

## 3. Discussion

This is the first study to prospectively evaluate the influence of baseline GLS in moderate- and high-risk patients on cancer therapy-related cardiac dysfunction. The main finding of this study is that baseline GLS less negative than −18% was a significant and independent risk factor for the development of CTRCD and all-cause mortality in the course of anthracycline-based cancer treatment, even after adjusting for other risk factors. The 2022 Cardio-oncology guidelines of the European Society of Cardiology recommend GLS monitoring for patients at risk of CTRCD. However, the guidelines do not currently include the use of baseline GLS assessment as a risk factor; instead, strain assessment is recommended solely to establish a baseline for measuring subsequent relative reduction in GLS. This study provides additional data supporting the consideration of decreased baseline GLS as an independent risk factor for CTRCD.

### 3.1. Impact of Baseline Left Ventricular GLS on CTRCD

Changes in GLS in patients receiving anthracycline-based chemotherapy are a well-established predictor of CTRCD, and a relative reduction of 15% or more is part of the definition of CTRCD [1]. The role of baseline GLS is not as well established, although other studies have suggested that it may serve as a predictor of CTRCD. However, there are significant differences between those studies and ours. A study by Kang et al. identified abnormal baseline GLS as one of the predictive factors of CTRCD in their risk score for patients with acute leukemia. The main difference lies in their lower cutoff value for baseline GLS, which was defined as >−15% [7]. Their findings were confirmed in our study, but with a higher baseline GLS cutoff, suggesting that even a smaller decrease in baseline GLS may be predictive of CTRCD. In the study by Hatazawa et al., even higher baseline GLS (≥−19%) was associated with LV dysfunction after anthracycline chemotherapy and hospitalization for HF during follow-up [8]. However, their cohort comprised older patients with lymphoma and demonstrated a significantly higher prevalence of Ann Arbor stage IV disease among those with left ventricular (LV) dysfunction compared with those without LV dysfunction. In a study by Charbonnel et al., baseline global longitudinal strain was significantly lower in patients who subsequently developed cardiotoxicity compared with controls. As this study was performed before new definitions of CTRCD were established, anthracycline cardiotoxicity was defined as a decrease in the LVEF of >10 percentage points, to a value <53% [9]. In study by Narayan et al. among longitudinal, circumferential, and radial peak systolic strain and strain rate measurements, circumferential strain was most strongly predictive of CTRCD [10]. However, at present only global longitudinal strain is recommended in cancer patients to diagnose and follow CTRCD. In a retrospective study by Araujo-Gutierrez et al., both decreased GLS and overall GLS were found to be predictive of CTRCD prior to anthracycline treatment [6]. The main difference between their study and ours is that our cohort consisted solely of moderate- and high-risk patients, whereas their study included only low-risk patients.

### 3.2. Impact of Decreased Baseline GLS on All-Cause Mortality

In the study by Araujo-Gutierrez et al., there was no significant difference in overall all-cause mortality between patients with decreased and normal baseline GLS; however, a difference was observed in cardiovascular-related mortality [6]. In contrast, the study by Kang et al. found that the all-cause mortality rate was significantly higher in patients with decreased GLS [7], a finding that was also confirmed in our study.

In our study, NT-proBNP was significantly associated with moderate/severe CTRCD in univariable analysis but did not remain an independent predictor in the multivariable model. Nevertheless, NT-proBNP remains a well-established biomarker of myocardial stress, and its integration into surveillance strategies should not be dismissed. Future studies should explore strategies that integrate decreased baseline GLS with established risk factors, as such a combined approach may improve early detection of cardiotoxicity, enable timely initiation of cardioprotective therapy, and ultimately optimize long-term cardiovascular outcomes in patients receiving potentially cardiotoxic cancer treatments.

In addition to highlighting the increased risk associated with decreased baseline GLS, it remains uncertain whether this risk can be reduced through cardioprotective strategies. Recent study has demonstrated that in patients with isolated GLS reduction following anthracycline treatment, cardioprotective therapy was linked to better preservation of LVEF over a one-year follow-up compared to standard care [11]. Future research should further investigate the potential benefits of cardioprotective interventions in patients with baseline GLS abnormalities. Our findings also align with the growing interest in multimodal approaches for cardiotoxicity risk prediction. Recent research published in Nature Communications (2024) demonstrated that AI-enabled models using baseline ECG can predict CTRCD risk. Combining such ECG-based models with echocardiographic parameters like baseline GLS could enhance early risk stratification [12].

Our findings align with and expand upon current recommendations from the 2022 European Society of Cardiology cardio-oncology guidelines and other international expert consensus statements, which endorse the use of GLS as a sensitive marker of subclinical myocardial dysfunction. While the guidelines emphasize the role of GLS for early detection of treatment-related cardiac dysfunction during therapy, our results suggest that its utility begins even earlier—at baseline assessment. Based on the findings of Araujo-Gutierrez et al. and our own study, it is reasonable to recommend that decreased baseline GLS (≥−18%) be considered a predictive marker for CTRCD in patients receiving anthracycline-based chemotherapy, regardless of their initial cardiovascular risk category. Incorporating baseline GLS into the pre-treatment risk assessment may enhance patient management by enabling the early initiation of cardioprotective strategies, even before a measurable decline in GLS occurs, which could ultimately improve clinical outcomes. Notably, the prognostic value of GLS appears particularly important in patients with a baseline LVEF above 54%, as those with LVEF ≤ 54% are already classified as being at risk. In our study, we found that among patients with baseline LVEF > 54%, a decreased baseline GLS was an independent and statistically significant risk factor for the development of CTRCD. Given that Araujo-Gutierrez et al. demonstrated similar results in low-risk patients, and our findings extend this association to moderate- and high-risk groups, decreased baseline GLS appears to be a robust prognostic marker for CTRCD irrespective of initial cardiovascular risk stratification. Nonetheless, prospective validation in larger and more diverse populations remains necessary.

### 3.3. Limitations

This study has several limitations. First, the population included a small cohort from a single cancer center, and the limited sample size may have reduced the statistical power to detect certain associations. This implies also that our study was not sufficiently powered to study the relative importance of other risk factors, including the known risk factors, in parallel with GLS reduction. Thus, the lack of significance for such predictors as the HFA-ICOS risk group or LVEF reduction in the multivariable analysis should not be interpreted as negative findings but rather as a consequence of a lack of statistical power, especially that the estimated effect sizes were high and they were significantly associated with CTRCD incidence in univariate analysis. Second, GLS was assessed by a single experienced cardiologist, and inter-observer and intra-observer variability was not evaluated. Third, manual entry of data into electronic case report forms carries a risk of transcription errors, although all entries were double-checked to minimize this possibility. Fourth, the follow-up period was limited to 12 months, preventing evaluation of late-onset CTRCD and long-term cardiovascular outcomes. Finally, the study population consisted almost exclusively of women, as most participants were being treated for breast cancer. This female predominance reflects the epidemiology of the underlying malignancy but limits the generalizability of our findings to male patients or to patients with other cancer types. Therefore, our results should be interpreted with caution when extrapolating to mixed-sex or male-dominated populations. Future multi-center studies with larger and more diverse populations, assessment of inter-observer reproducibility, and extended follow-up are warranted to validate and expand on our findings.

## 4. Materials and Methods

### 4.1. Study Structure

The ANTEC study was a single-center prospective observational trial that evaluates the impact of atherosclerosis in the coronary arteries and the coronary artery calcium score determined by computed tomography on the risk of CTRCD related to anthracycline-based chemotherapy. The trial was carried out according to the principles of the Declaration of Helsinki and the guidelines of the International Conference on Harmonization Good Clinical Practice. The conduct of the study was approved by an Independent Ethics Committee at the Maria Sklodowska-Curie National Research Institute of Oncology in Warsaw (date of approval: 28 October 2021, approval no. 80/2021) and all participants provided written informed consent before study entry. The registration identifier on clinicaltrials.gov is NCT05118178.

**Figure 3:**
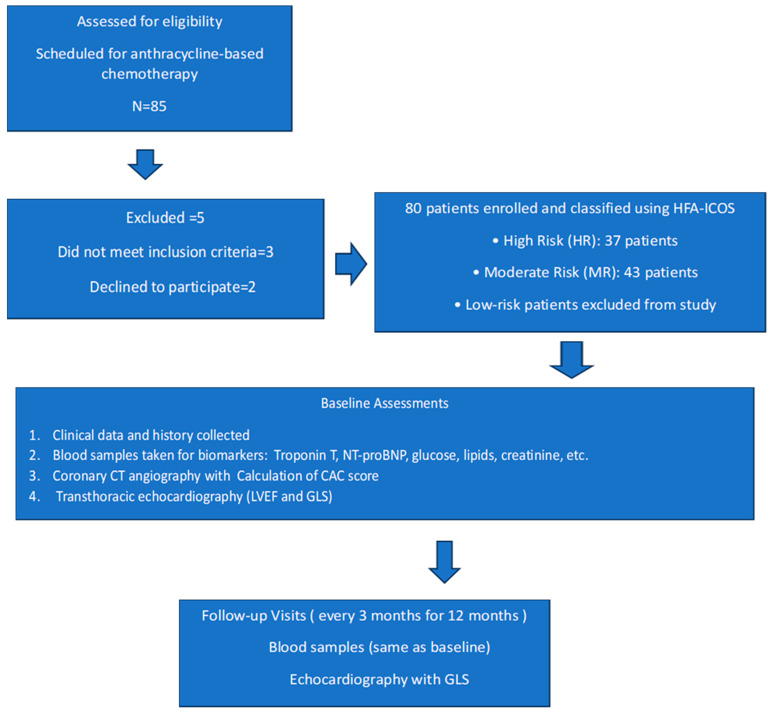
Study flowchart.

The study enrolled 80 cancer patients who were stratified as moderate risk (MR) or high risk (HR) of cancer therapy-related cardiovascular toxicity according to the Heart Failure Association-International Cardio-Oncology Society baseline cardiovascular toxicity risk stratification model [13], diagnosed and qualified for systemic treatment with anthracycline chemotherapy at the Maria Sklodowska-Curie National Research Institute of Oncology in Warsaw. Patients were enrolled between November 2021 and September 2023, with a follow-up period of 12 months. The study protocol with inclusion and exclusion criteria, schedule visits, and procedures was published previously [14]. Low-risk patients were excluded from the study. No patients had a prior history of chemotherapy, radiotherapy, coronary artery disease, or heart failure at the time of study enrolment. Flowchart for the study is presented in Figure 3.

### 4.2. Patient Classification and Echocardiographic Assessment

Clinical data were obtained from patients’ medical records and medical history. All participants underwent comprehensive transthoracic echocardiography using a Philips EPIQ Elite system (Philips Healthcare, Andover, MA, USA). Standard two-dimensional, M-mode, and Doppler images were acquired in accordance with the current recommendations of the American Society of Echocardiography (ASE) and the European Association of Cardiovascular Imaging (EACVI). The echocardiographic assessment included measurement of ventricular dimensions, systolic and diastolic function, and two-dimensional left ventricular peak systolic global longitudinal strain (GLS). For strain analysis, endocardial borders were manually traced in three standard apical views (four-, two-, and three-chamber). GLS was calculated by measuring the total endocardial contour length at end-diastole and end-systole in each view, and the final value was derived as the average across all three views. Each parameter was averaged over three consecutive cardiac cycles, with frame rates maintained between 50 and 90 frames per second to ensure optimal speckle tracking. All echocardiographic analyses were performed by a single board-certified cardiologist blinded to patients’ clinical data. Decreased baseline left ventricular GLS was defined as ≥−18% (less negative than −18%), according to current ASE/EACVI consensus recommendations [15,16].

Blood samples were collected and analyzed at the central laboratory of the Maria Sklodowska-Curie National Research Institute of Oncology. Laboratory assessments included creatinine, glucose, complete blood count, glycated hemoglobin, lipid profile, and plasma concentrations of troponin T (TnT) and N-terminal pro–B-type natriuretic peptide (NT-proBNP).

### 4.3. Primary Endpoint

The primary endpoint was the appearance of cancer therapy-related cardiac dysfunction defined as mild, moderate, severe, or very severe according to the ESC 2022 Cardio-oncology guidelines at any time during follow-up [1]. The use of these contemporary, consensus-based criteria was chosen to standardize outcome classification, minimize heterogeneity in diagnosis, and enhance comparability of our results with other recent studies and current clinical practice.

### 4.4. Secondary Endpoint

The secondary endpoint reported in the current study was an all-cause mortality within 12 months follow-up.

### 4.5. Data Acquisition and Statistical Analysis

#### 4.5.1. Data Acquisition and Quality Control

All measurements were performed according to routine diagnostic standards. Critical measurements were reviewed and coded by a trained member of the study team to ensure comparability of the results. Clinical data was captured by manual entry into electronic case report forms (eCRFs) and laboratory data extracted from the Hospital Information System (HIS). The data was validated by the clinical team to eliminate inconsistent, missing, and outlying values.

#### 4.5.2. Sample Size Calculations and Statistical Analysis

The sample size was calculated for the primary objective of the study, i.e., the predictive value of coronary atherosclerosis for CTRCD incidence [14]. In the current study we evaluate secondary objective, the impact of baseline GLS on incidence of CTRCD. To understand the study sample size adequacy, we performed calculations assuming that approximately 1/3 of the patients will have decreased baseline GLS (≥−18%), and that the risk ratio will be 3.5, taking into consideration the results of previous study [6]. We further assumed the incidence of CTRCD of 0.12 as observed in low-risk group as a conservative estimate of the incidence in patients with normal GLS [6]. With additional standard assumptions of 0.05 statistical significance and 80% power and assuming that the estimated sample size necessary for our objective would be 69 individuals, indicating that the effective sample size of the original study (77 individuals) is likely to be sufficient. Descriptive statistics including percent distribution for categorical variables and mean, standard deviation (sd) and percentiles for numerical variables are reported. The chi-square and Fisher exact tests for categorical and non-parametric Wilcoxon rank-sum test (due to concerns of skewedness of the distributions) for numerical variables were used to compare the demographic, clinical and laboratory characteristics in groups determined on the basis of the presence of decreased GLS. Log-binomial multivariable regression was used to study independent risk factors associated with occurrence of moderate-to-severe CTRCD during the follow up period. Multivariable model was purposefully selected to include established risk factors of CTRCD. Significant results are reported at the alfa = 0.05 level. Statistical analysis was performed in Stata/SE 17.0 (StataCorp LCC, College Station, TX, USA).

## 5. Conclusions

Among the parameters studied, decreased left ventricular global longitudinal strain was the only significant and independent predictor of cancer therapy-related cardiac dysfunction in moderate- and high-risk patients receiving anthracycline-based therapy. Moreover, patients with decreased baseline GLS demonstrated a significantly higher all-cause mortality rate. These findings support the potential incorporation of baseline GLS into pre-treatment cardiovascular risk assessment, particularly in patients with a left ventricular ejection fraction above 54%, to better identify individuals who may benefit from early cardioprotective interventions. Given that Araujo-Gutierrez et al. reported similar findings in low-risk patients, and our results extend this association to higher-risk populations, decreased baseline GLS appears to be a robust prognostic marker for CTRCD regardless of initial cardiovascular risk stratification.

## Figures and Tables

**Table 1:** Characteristics of the study sample, by normal vs. decreased GLS.

Factor		Normal GLS, *n* = 56	Decreased GLS, *n* = 21	Total, *n* = 77	*p*-Values
Age group (years)	<65	33 (58.9%)	8 (38.1%)	41 (53.2%)	0.10
	≥65	23 (41.1%)	13 (61.9%)	36 (46.8%)	
Cancer	breast	42 (75.0%)	17 (81.0%)	59 (76.6%)	0.72
	lymphoma	6 (10.7%)	1 (4.8%)	7 (9.1%)	
	sarcoma	8 (14.3%)	3 (14.3%)	11 (14.3%)	
Risk group	High	24 (42.9%)	13 (61.9%)	37 (48.1%)	0.14
	Moderate	32 (57.1%)	8 (38.1%)	40 (51.9%)	
BMI (kg/m^2^)	<30	40 (71.4%)	14 (66.7%)	54 (70.1%)	0.68
	≥30	16 (28.6%)	7 (33.3%)	23 (29.9%)	
SBP (mmHg)-mean (sd)		137.1 (14.0)	140.4 (18.1)	138.0 (15.2)	0.36
DBP (mmHg)-mean (sd)		83.6 (7.8)	84.1 (10.5)	83.7 (8.5)	0.53
NYHA scale	1	42 (75.0%)	12 (57.1%)	54 (70.1%)	0.13
	2	14 (25.0%)	9 (42.9%)	23 (29.9%)	
ECOG score	0	43 (76.8%)	12 (57.1%)	55 (71.4%)	0.09
	1	13 (23.2%)	8 (38.1%)	21 (27.3%)	
	2	0 (0.0%)	1 (4.8%)	1 (1.3%)	
Coexisting conditions	Hypertension	41 (73.2%)	19 (90.5%)	60 (77.9%)	0.10
	Hyperlipidemia	41 (73.2%)	17 (81.0%)	58 (75.3%)	0.48
	Ever smoking	26 (46.4%)	11 (52.4%)	37 (48.1%)	0.64
	Diabetes	8 (14.3%)	3 (14.3%)	11 (14.3%)	0.99
	Chronic kidney disease	8 (14.3%)	4 (19.0%)	12 (15.6%)	0.61
Concomitant medications	Beta-blockers	23 (41.1%)	10 (47.6%)	33 (42.9%)	0.61
	ACE-I	21 (37.5%)	13 (61.9%)	34 (44.2%)	0.06
	Statins	15 (26.8%)	8 (38.1%)	23 (29.9%)	0.33
Anthracycline dose (mg/m^2^)	<250	43 (76.8%)	19 (90.5%)	62 (80.5%)	0.18
	≥250	13 (23.2%)	2 (9.5%)	15 (19.5%)	
Troponin T (ng/L)	≤14	52 (92.9%)	19 (90.5%)	71 (92.2%)	0.73
	>14	4 (7.1%)	2 (9.5%)	6 (7.8%)	
NT-proBNP (pg/mL)	≤125	39 (69.6%)	9 (42.9%)	48 (62.3%)	0.031
	>125	17 (30.4%)	12 (57.1%)	29 (37.7%)	
LVEF (%)-mean (sd)		62.2 (2.9)	60.0 (4.5)	61.6 (3.5), 62 (50–69)	0.07

**Table 2:** Characteristics of patients with moderate/severe CTRCD vs. No/mild CTRCD groups (*n* = 77).

Factor		No/Mild CTRCD, *n* = 67	Moderate/Severe CTRCD, *n* = 10	Total, *n* = 77	*p*-Values
Age group (years)	<65	38 (56.7%)	3 (30.0%)	41 (53.2%)	0.11
	≥65	29 (43.3%)	7 (70.0%)	36 (46.8%)	
Age (years), mean		59.7 (12.5)	65.2 (9.4)	60.5 (12.2)	0.18
Cancer	breast	52 (77.6%)	7 (70.0%)	59 (76.6%)	0.42
	lymphoma	5 (7.5%)	2 (20.0%)	7 (9.1%)	
	sarcoma	10 (14.9%)	1 (10.0%)	11 (14.3%)	
Risk group	HR	29 (43.3%)	8 (80.0%)	37 (48.1%)	0.03
	MR	38 (56.7%)	2 (20.0%)	40 (51.9%)	
BMI (kg/m^2^)	<30	49 (73.1%)	5 (50.0%)	54 (70.1%)	0.14
	≥30	18 (26.9%)	5 (50.0%)	23 (29.9%)	
BMI (kg/m^2^), mean		27.6 (4.7)	29.6 (8.0)	27.9 (5.2)	0.59
NYHA scale	1	48 (71.6%)	6 (60.0%)	54 (70.1%)	0.45
	2	19 (28.4%)	4 (40.0%)	23 (29.9%)	
ECOG score	0	49 (73.1%)	6 (60.0%)	55 (71.4%)	0.59
	1	17 (25.4%)	4 (40.0%)	21 (27.3%)	
	2	1 (1.5%)	0 (0.0%)	1 (1.3%)	
Coexisting conditions:					
Hypertension	Yes	51 (76.1%)	9 (90.0%)	60 (77.9%)	0.32
Hyperlipidemia	Yes	50 (74.6%)	8 (80.0%)	58 (75.3%)	0.71
Ever smoking	Yes	33 (49.3%)	4 (40.0%)	37 (48.1%)	0.59
Diabetes	Yes	10 (14.9%)	1 (10.0%)	11 (14.3%)	0.68
Chronic kidney disease	Yes	10 (14.9%)	2 (20.0%)	12 (15.6%)	0.68
Medications:					
Beta-blockers	Yes	27 (40.3%)	6 (60.0%)	33 (42.9%)	0.24
ACE-I	Yes	28 (41.8%)	6 (60.0%)	34 (44.2%)	0.28
Statins	Yes	18 (26.9%)	5 (50.0%)	23 (29.9%)	0.14
Anthracycline dose (mg/m^2^)	<250	54 (80.6%)	8 (80.0%)	62 (80.5%)	0.97
	≥250	13 (19.4%)	2 (20.0%)	15 (19.5%)	
Anthracycline dose (mg/m^2^), median [IQR]		240.0 [240.0–240.0]	240.0 [240.0–240.0]	240.0 [240.0–240.0]	0.80
Troponin T (ng/L)	≤14	62 (92.5%)	9 (90.0%)	71 (92.2%)	0.78
	>14	5 (7.5%)	1 (10.0%)	6 (7.8%)	
Troponin T (ng/L), median [IQR]		6.6 [5.2–10.0]	8.9 [7.2–10.6]	7 [5.4–10]	0.06
NT-proBNP (pg/mL)	≤125	44 (65.7%)	4 (40.0%)	48 (62.3%)	0.12
	>125	23 (34.3%)	6 (60.0%)	29 (37.7%)	
NT-proBNP (pg/mL), median [IQR]		84.0 [36.4–156.0]	196.5 [49.0–399.0]	94 [38.5–180]	0.10
GLS (%)	<−18%	54 (80.6%)	2 (20.0%)	56 (72.7%)	<0.001
	≥−18%	13 (19.4%)	8 (80.0%)	21 (27.3%)	
GLS (%), mean		19.4 (1.9)	17.1 (2.1)	19.1 (2.0)	0.002
LVEF (%), mean		62.0 (3.0)	59.0 (5.3)	61.6 (3.5)	0.11

**Table 3:** Univariate and multivariate risk ratios of moderate/severe CTRCD.

Factor		Univariate Risk Ratio for Moderate/Severe CTRCD, 95% CI	Univariable *p*-Value	Multivariable Risk Ratio for Moderate/Severe CTRCD, 95% CI	Multivariable *p*-Value
Sex	Female	Ref.		Ref.	
	Male	1.6 (0.25–10.2)	0.62	0.22 (0.02–2.7)	0.23
Age group	<65	Ref.		Ref.	
	≥65	2.7 (0.7–9.5)	0.13	2.1 (0.31–14.8)	0.44
Age (years)	per year increase	1.05 (0.98–1.10)	0.98	-	
Risk group	HR	4.3 (1.0–19.1)	0.05	3.9 (0.4–35.8)	0.23
	MR	Ref.		Ref.	
BMI (kg/m^2^)	<30	Ref.		Ref.	
	≥30	2.3 (0.8–7.3)	0.14	2.3 (0.7–7.7)	0.20
Hypertension	No	Ref.		Ref.	
	Yes	2.5 (0.3–18.7)	0.36	0.3 (0.03–3.9)	0.39
Hyperlipidemia	No	Ref.		Ref.	
	Yes	1.3 (0.3–5.6)	0.72	0.6 (0.09–3.7)	0.57
Ever smoking	No	Ref.		Ref.	
	Yes	0.7 (0.2–2.4)	0.59	0.4 (0.08–2.3)	0.33
Diabetes	No	Ref.		Ref.	
	Yes	0.7 (0.1–4.8)	0.69	1.8 (0.22–14.2)	0.59
Chronic kidney disease	No	Ref.		Ref.	
	Yes	1.4 (0.3–5.6)	0.68	0.3 (0.03–2.4)	0.24
Troponin T (ng/L)	≤14	Ref.		Ref.	
	>14	1.3 (0.2–8.7)	0.78	0.6 (0.07–5.3)	0.64
Troponin T (ng/L)	per unit increase on log scale	2.8 (0.8–9.5)	0.10	-	
NT-proBNP (pg/mL)	≤125	Ref.		Ref.	
	>125	2.5 (0.8–8.1)	0.13	0.5 (0.1–2.0)	0.32
NT-proBNP (pg/mL)	per unit increase on log scale	1.8 (1.0–3.3)	0.047		
GLS (%)	<−18%	Ref.		Ref.	
	≥−18%	10.7 (2.5–46.2)	0.002	12.0 (2.0–71.9)	0.007
GLS (%)	per unit increase	0.58 (0.49–0.68)	<0.001		
LVEF (%)	≥55%	Ref.		Ref.	
	50–54%	7.8 (3.2–19.3)	<0.001	4.5 (0.7–28.5)	0.11
LVEF (%)	per unit increase	0.82 (0.77–0.87)	<0.001		

## Data Availability

The original contributions presented in this study are included in the article. Further inquiries can be directed to the corresponding author.

## Abbreviations

CTRCD	cancer therapy-related cardiac dysfunction
CTR-CVT	cancer therapy-related cardiovascular toxicity
HFA-ICOS	Heart Failure Association-International Cardio-Oncology Society
GLS	left ventricular peak systolic global longitudinal strain
hs-cTnT	high-sensitivity cardiac troponin T
NT-proBNP	N-terminal pro-B-type natriuretic peptide
eCRF	electronic case report forms
HIS	Hospital Information System
LVEF	left ventricle ejection fraction
CVD	cardiovascular disease

## References

[1] Lyon A.R., Lopez-Fernandez T., Couch L.S., Asteggiano R., Aznar M.C., Bergler-Klein J., Boriani G., Cardinale D., Cordoba R., Cosyns B. (2022). 2022 ESC Guidelines on cardio-oncology developed in collaboration with the European Hematology Association (EHA), the European Society for Therapeutic Radiology and Oncology (ESTRO) and the International Cardio-Oncology Society (IC-OS). Eur. Heart J. Cardiovasc. Imaging.

[2] Negishi K., Negishi T., Hare J.L., Haluska B.A., Plana J.C., Marwick T.H. (2013). Independent and incremental value of deformation indices for prediction of trastuzumab-induced cardiotoxicity. J. Am. Soc. Echocardiogr..

[3] Oikonomou E.K., Kokkinidis D.G., Kampaktsis P.N., Amir E.A., Marwick T.H., Gupta D., Thavendiranathan P. (2019). Assessment of Prognostic Value of Left Ventricular Global Longitudinal Strain for Early Prediction of Chemotherapy-Induced Cardiotoxicity: A Systematic Review and Meta-analysis. JAMA Cardiol..

[4] Stoodley P.W., Richards D.A., Boyd A., Hui R., Harnett P.R., Meikle S.R., Byth K., Stuart K., Clarke J.L., Thomas L. (2013). Left ventricular systolic function in HER_2_/neu negative breast cancer patients treated with anthracycline chemotherapy: A comparative analysis of left ventricular ejection fraction and myocardial strain imaging over 12 months. Eur. J. Cancer.

[5] Thavendiranathan P., Poulin F., Lim K.D., Plana J.C., Woo A., Marwick T.H. (2014). Use of myocardial strain imaging by echocardiography for the early detection of cardiotoxicity in patients during and after cancer chemotherapy: A systematic review. J. Am. Coll. Cardiol..

[6] Araujo-Gutierrez R., Chitturi K.R., Xu J., Wang Y., Kinder E., Senapati A., Chebrolu L.B., Kassi M., Trachtenberg B.H. (2021). Baseline global longitudinal strain predictive of anthracycline-induced cardiotoxicity. Cardio-Oncology.

[7] Kang Y., Assuncao B.L., Denduluri S., McCurdy S., Luger S., Lefebvre B., Carver J., Scherrer-Crosbie M. (2019). Symptomatic Heart Failure in Acute Leukemia Patients Treated with Anthracyclines. JACC CardioOncol..

[8] Hatazawa K., Tanaka H., Nonaka A., Takada H., Soga F., Hatani Y., Matsuzoe H., Shimoura H., Ooka J., Sano H. (2018). Baseline Global Longitudinal Strain as a Predictor of Left Ventricular Dysfunction and Hospitalization for Heart Failure of Patients with Malignant Lymphoma After Anthracycline Therapy. Circ. J..

[9] Charbonnel C., Convers-Domart R., Rigaudeau S., Taksin A.L., Baron N., Lambert J., Ghez S., Georges J.-L., Farhat H., Lambert J. (2017). Assessment of global longitudinal strain at low-dose anthracycline-based chemotherapy, for the prediction of subsequent cardiotoxicity. Eur. Heart J. Cardiovasc. Imaging.

[10] Narayan H.K., French B., Khan A.M., Plappert T., Hyman D., Bajulaiye A., Domchek S., DeMichele A., Clark A., Matro J. (2016). Noninvasive Measures of Ventricular-Arterial Coupling and Circumferential Strain Predict Cancer Therapeutics–Related Cardiac Dysfunction. JACC Cardiovasc. Imaging.

[11] Marwick T.H., Dewar E., Nolan M., Shirazi M., Dias P., Wright L., Fitzgerald B., Kearney L., Srivastava P., Atherton J. (2024). Strain surveillance during chemotherapy to improve cardiovascular outcomes: The SUCCOUR-MRI trial. Eur. Heart J..

[12] Yagi R., Goto S., Himeno Y., Katsumata Y., Hashimoto M., MacRae C.A., Deo R.C. (2024). Artificial intelligence-enabled prediction of chemotherapy-induced cardiotoxicity from baseline electrocardiograms. Nat. Commun..

[13] Herrmann J., Lenihan D., Armenian S., Barac A., Blaes A., Cardinale D., Carver J., Dent S., Ky B., Lyon A.R. (2022). Defining cardiovascular toxicities of cancer therapies: An International Cardio-Oncology Society (IC-OS) consensus statement. Eur. Heart J..

[14] Borowiec A., Ozdowska P., Rosinska M., Jagiello-Gruszfeld A., Jasek S., Waniewska J., Kotowicz B., Kosela-Paterczyk H., Lampka E., Makowka A. (2023). Prognostic value of coronary atherosclerosis and CAC score for the risk of chemotherapy-related cardiac dysfunction (CTRCD): The protocol of ANTEC study. PLoS ONE.

[15] Liu J.E., Barac A., Thavendiranathan P., Scherrer-Crosbie M. (2020). Strain Imaging in Cardio-Oncology. JACC CardioOncol..

[16] Thomas J.D., Edvardsen T., Abraham T., Appadurai V., Badano L., Banchs J., Cho G.Y., Cosyns B., Delgado V., Donal E. (2025). Clinical Applications of Strain Echocardiography: A Clinical Consensus Statement From the American Society of Echocardiography Developed in Collaboration with the European Association of Cardiovascular Imaging of the European Society of Cardiology. J. Am. Soc. Echocardiogr..

